# Effects of Active Video Games on Health-Related Physical Fitness and Motor Competence in Children and Adolescents With Overweight or Obesity: Systematic Review and Meta-Analysis

**DOI:** 10.2196/29981

**Published:** 2021-10-18

**Authors:** Cristina Comeras-Chueca, Jorge Marin-Puyalto, Angel Matute-Llorente, German Vicente-Rodriguez, Jose Antonio Casajus, Alex Gonzalez-Aguero

**Affiliations:** 1 Department of Physiatry and Nursing Faculty of Health Science University of Zaragoza Zaragoza Spain; 2 GENUD Research Group (Growth, Exercise, NUtrition and Development) University of Zaragoza Zaragoza Spain; 3 EXERNET Red de Investigación en Ejercicio Físico y Salud para Poblaciones Especiales Zaragoza Spain; 4 Department of Physiatry and Nursing Faculty of Health and Sport Science University of Zaragoza Huesca Spain; 5 Instituto Agroalimentario de Aragón -IA2- CITA Universidad de Zaragoza Zaragoza Spain; 6 Centro de Investigación Biomédica en Red de Fisiopatología de la Obesidad y Nutrición (CIBERObn) Madrid Spain

**Keywords:** active videogames, exergaming, BMI, body fat, motor skills, cardiorespiratory fitness, muscle

## Abstract

**Background:**

Childhood obesity is one of the most important public health problems. Active video games (AVGs) have been proposed as an attractive alternative to increase energy expenditure and are being investigated to determine their effectiveness against childhood obesity.

**Objective:**

The aim of this study is to summarize the existing research and draw conclusions about the effects of AVGs on health-related physical fitness and motor competence in children and adolescents with overweight and obesity.

**Methods:**

The search strategy was applied to PubMed, MEDLINE, Web of Science, and SPORTDiscus, including randomized and nonrandomized controlled trials investigating the effects of AVG programs on health-related physical fitness and motor competence in children and adolescents with overweight and obesity. To measure the risk of bias in randomized and nonrandomized controlled trials, 2 different quality assessment tools were used. In total, 15 articles met the inclusion criteria, and the variables of interest were BMI, body fat percentage, cardiorespiratory fitness (CRF), waist circumference, fat-free mass, muscular fitness, and motor competence. A meta-analysis was performed.

**Results:**

Positive effects were found for BMI and body fat percentage, favoring the AVG group compared with a control group with no intervention (mean difference −0.209; 95% CI −0.388 to −0.031 vs mean difference −0.879; 95% CI −1.138 to −0.602). Positive effects seem to be observed for CRF. The effects of AVG interventions on muscular fitness, fat-free mass, waist circumference, and motor competence are unclear.

**Conclusions:**

AVG programs showed positive effects on BMI, body fat percentage, and CRF. AVG could be a good strategy to combat childhood obesity.

## Introduction

### Background

Childhood obesity is one of the most important public health problems in the 21st century in high-income societies [1]. The prevalence of overweight and obesity in childhood has acquired the status of an epidemic. The global prevalence of overweight and obesity among children and adolescents (aged 5-19 years) has risen dramatically from 4% in 1975 to over 18% in 2016. For instance, the prevalence of overweight was over 30%, and the prevalence of obesity was over 10% in European children and adolescents in 2016 [2]. Obesity has become a pandemic owing to an obesogenic environment that causes cardiovascular and cardiometabolic diseases and psychosocial problems [3,4]. Children with overweight and obesity are likely to remain obese during adulthood and are more likely to develop many other types of cardiovascular and metabolic pathologies [1]. Evidence shows that cardiovascular risk is inversely related to physical fitness [5] and the amount of physical activity (PA) [6] performed by youth. The components of health-related physical fitness are cardiorespiratory fitness (CRF), body composition, muscular strength, muscular endurance, and flexibility [7]. Childhood obesity is related to poor health-related physical fitness, such as CRF and muscular strength [8].

On the other hand, the recommendation of the World Health Organization indicates that a daily average of 60 minutes of moderate-to-vigorous PA provides any of the health benefits in young people, although daily average of beyond 60 minutes of moderate-to-vigorous PA provides additional benefits [9]. In 2016, a study including 1.6 million students aged 11-17 years showed that 81% of them did not meet this recommendation [10]. PA, especially at moderate-to-vigorous intensity, is associated with better physical fitness, independent of sedentary time [11,12].

In addition, one of the main sedentary behaviors of this population is playing electronic games, such as computer or console games [13]. The World Health Organization reported that 40.2% of children and adolescents spend at least 2 hours per day watching television or using electronic devices on weekdays, and this percentage rises to 75.8% during weekends, going further than the recommendations of maximum screen time [14]. This inactivity and excessive sedentary screen time are catastrophic for motor development in children and adolescents [15]. A recent systematic review performed by Han et al [16] showed that children and adolescents with overweight and obesity have a lower motor competence level than children and adolescents with healthy weight; therefore, low motor competence needs to be taken into consideration in children with overweight or obesity. Moreover, children with high actual and perceived motor competence will probably show higher PA and lower BMI status [17]. An improvement in motor competence may promote better perceived motor competence, which entails higher motivation and participation in extracurricular PA and sports [18,19]. In addition, evidence shows a relationship between motor competence and health-related physical fitness during childhood and adolescence [20]. Thus, improving motor skills in children with overweight and obesity is one of the main objectives.

It is well known that exercise is an effective tool to fight obesity [21], with all its associated benefits, such as improvements in BMI status or adiposity, cardiorespiratory and muscular fitness, or bone health [22]. However, the main challenge is to ensure adherence to exercise in children with overweight and obesity [23]. Therefore, the implementation of new types of exercise that are more attractive and motivational to this population is needed.

Active video games (AVGs) have been proposed as a suitable alternative to exercise and are being investigated to determine their effectiveness against childhood obesity. AVGs generally require full-body movement and therefore increase energy expenditure [24]. A systematic review showed that structured AVG sessions had the potential to increase PA in children, but there was no evidence of the benefits of conducting them in the home setting [25]. PA and energy expenditure during AVGs are a well-studied topic showing that AVGs elicit light-to-moderate PA, and also elevate energy expenditure to moderate-to-vigorous intensity, thus having a favorable influence on energy balance [26-28]. Nevertheless, energy expenditure has been found to be higher in structured programs [29]. AVGs seem to be an interesting strategic tool to encourage an active and healthy lifestyle as an alternative to sedentary behaviors [30-32]. However, according to the overview performed by Kari [33], additional high-quality research and systematic reviews concerning exergaming are needed. In addition, AVGs seem to be an effective tool for improving self-concept, self-efficacy, situational interest and motivation, enjoyment, and psychological and social well-being [34,35]. Specifically, AVGs may have positive effects on the psychological aspects and mental health of children and adolescents with overweight or obese [36,37].

Finally, AVGs may have a positive effect on motor competence and health-related physical fitness. Some studies have shown enhancements in children’s motor competence and perceived competence [38-40] or improvements in health-related physical fitness, such as cardiorespiratory and muscular fitness [41-43] and body composition [44], after an AVG intervention.

### Objective

Therefore, the main aim of this systematic review is to summarize and critically appraise the existing research on the effects of AVGs on health-related physical fitness and motor competence in children and adolescents with overweight and obesity and to extract conclusions from a fair comparison of the studies included.

## Methods

### Data Sources and Search Strategy

This review was performed following the criteria and methodology established by the Cochrane Handbook for Systematic Reviews of Interventions (version 5.1.0) [45]. This review was performed according to the PRISMA (Preferred Reporting Items for Systematic reviews and Meta-Analyses) 2020 statement [46]. The PRISMA checklist is shown in Multimedia Appendix 1. The protocol was registered in the International Prospective Register of Systematic Reviews, PROSPERO (CRD42020189138).

Journal articles were identified by searching electronic databases, scanning reference lists of articles, and examining tables from previous systematic reviews. The search strategy was applied to PubMed, MEDLINE, Web of Science, and SPORTDiscus up to and including March 2021.

The search strategy used to identify the articles in PubMed and MEDLINE was as follows: exergam* OR active video gam* OR active videogam* OR active gam* OR interactive video gam* OR interactive videogam* OR *Wii* OR *Xbox* OR *Kinect* OR *PlayStation*, and *Species: Humans* and *Language: English* filters were applied, along with *Journal Article* for MEDLINE. The search strategy applied in SPORTDiscus was as follows: TX=(exergam* OR active gam* OR active video gam* OR active videogam* OR interactive video gam* OR interactive videogam* OR *Wii* OR *Xbox* OR *Kinect* OR *PlayStation*) and *document type: article* and *language: English* filters were applied. The search strategy used in Web of Science was as follows: TS=(exergam* OR active gam* OR active video gam* OR active videogam* OR interactive video gam* OR interactive videogam* OR *Wii* OR *Xbox* OR *Kinect* OR *PlayStation*) and *document type: article* and *language: English* filters were applied.

Two reviewers (CCC and AGA) independently evaluated all studies. Titles and abstracts were examined, and relevant articles were obtained and assessed using the inclusion and exclusion criteria presented in Textbox 1. The inclusion criteria were used following the PICOS (Population, Intervention, Comparison, Outcomes and Study) format [47]. Interreviewer disagreements were resolved by consensus. A third reviewer (JAC) resolved these disagreements.

**Inclusion and exclusion criteria.**

**Inclusion criteria**

- Types of participants were children and adolescents with overweight and obesity
- Trials studying the effects of active video game programs on health-related physical fitness and motor competence
- Control group with no intervention or with traditional exercise intervention
- Types of outcome measures included variables of health-related physical fitness, such as cardiorespiratory fitness, musculoskeletal fitness (muscular strength and muscular endurance), and body composition and variables related to motor competence
- Types of studies were randomized and nonrandomized controlled trials

**Exclusion criteria**

- Studies were conducted in languages other than English or Spanish
- Data were unpublished
- Studies were conducted with animals
- Studies included participants aged ≥18 years
- Studies included participants with disabilities, diseases, or disorders other than obesity
- Studies were conducted without pre- and postassessments of the variables of interest
- Studies were dissertations or abstracts from society proceedings or congresses
- Studies included participants with normal weight
- Noncontrolled trials were considered in the discussion of the article with the consideration of the great limitation of the lack of a control group in interpretation of the results
- Noncontrolled trials were not included in the risk of bias assessments or meta-analysis
- All the noncontrolled trials concerning the effects of active video games on motor competence and health-related physical fitness in children and adolescents with overweight and obesity are summarized in Multimedia Appendix 2

### Risk of Bias

For assessing risk of bias proposed in the PRISMA 2020 statement, 2 risk of bias assessment tools were used—the Risk of Bias 2 in randomized controlled trials (RCTs) updated by Sterne et al [48] and the ROBINS-I (Risk of Bias in Nonrandomised Studies of Interventions) in nonrandomized controlled trials developed by Sterne et al [49].

### Data Extraction

The following information was extracted from each included trial: name of first author, year of publication, sample size, participant characteristics including number of participants, age and sex, type of study, type of intervention, training characteristics including intervention length and frequency, variables and data sources, and outcomes. The reported variables were weight, BMI, z-score of BMI, fat mass, body fat percentage, CRF, waist circumference, fat-free mass, muscular fitness, and motor competence.

### Meta-Analyses

Children and adolescents with overweight and obesity who underwent an AVG intervention were compared with a control group (ie, group with participants performing a PA intervention and with nonintervention participants). Effect sizes were calculated for each outcome (BMI, body fat percentage, CRF, waist circumference, fat-free mass, muscular fitness, and motor competence). Different meta-analyses were performed by stratifying the studies by type of control group (no intervention or exercise intervention without AVG). When the number of articles made it possible, analyses by subgroups were performed by dividing the studies by the length of the intervention. The free cross-platform software OpenMeta[Analyst] for advanced meta-analysis was used for data processing.

Mean differences (MD) between participants in AVG interventions and controls and their 95% CIs were calculated using a continuous random-effects model (DerSimonian-Laird method). The heterogeneity of the studies was tested using the I² statistic [48]. This statistic describes the variance between studies as a proportion of the total variance and was interpreted as follows: I^2^=0%-25% no heterogeneity; I^2^=25%-50% moderate heterogeneity; I^2^=50%-75% high heterogeneity; and I^2^=75%-100% very high heterogeneity. All analyses were performed using the OpenMeta[Analyst] software.

## Results

### Search Summary

**Figure 1:**
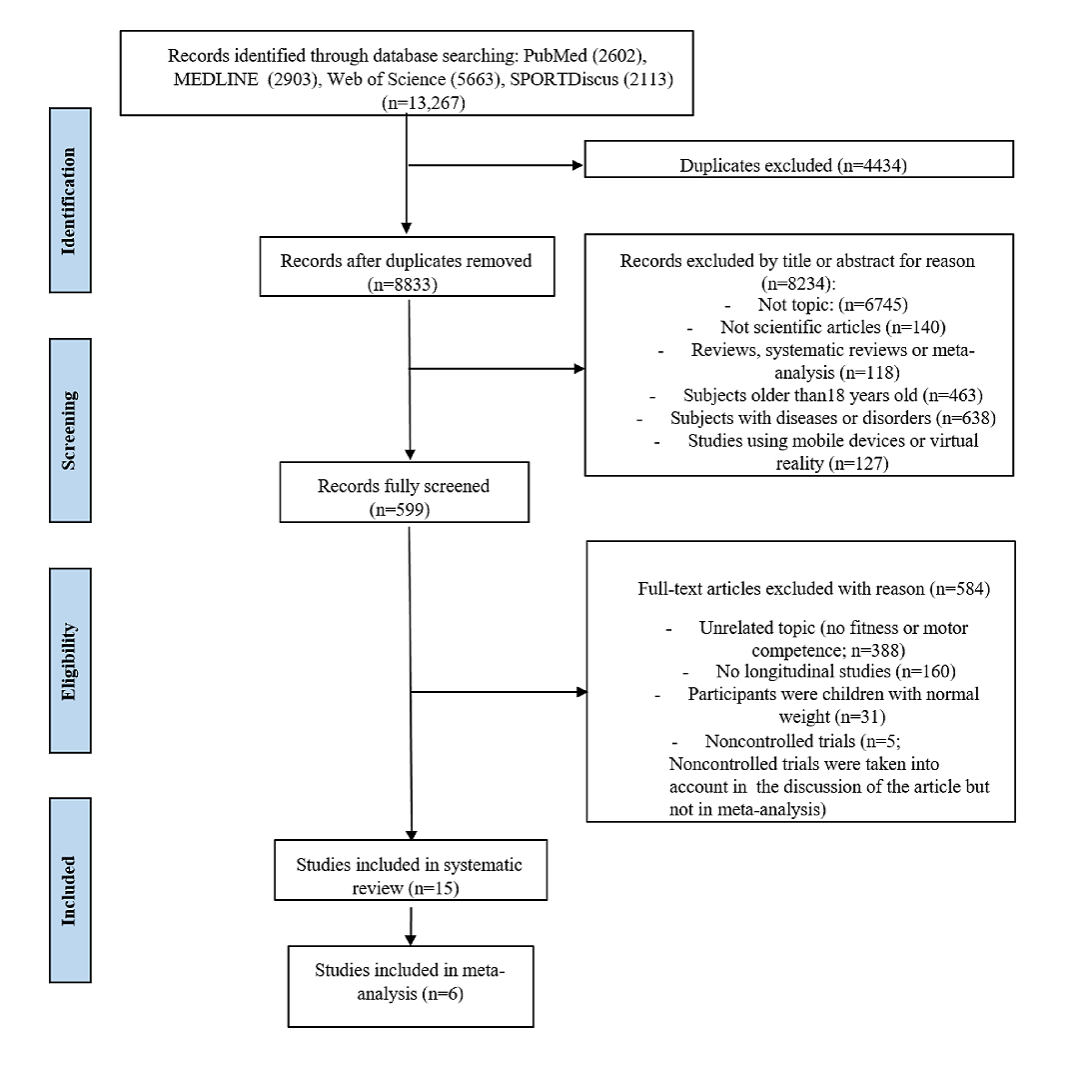
PRISMA (Preferred Reporting Items for Systematic Reviews and Meta-Analyses) flow diagram of articles that were selected.

A total of 13,267 relevant articles were identified using the abovementioned search strategies. Following a review of titles and abstracts and excluding duplicates, the total number of articles was reduced to 599. Of them, 15 articles met the inclusion criteria and were selected for this review. Articles were excluded for the following reasons: studies were cross-sectional (n=160); only psychological, cognitive, nutritional, and balance variables, PA, or energy expenditure were measured (n=388); participants were children with normal weight (n=31); and studies were noncontrolled trials (n=5; Figure 1).

The characteristics of each study included in this systematic review were summarized in different sections following the PICOS format [47].

### Methodological Quality

Multimedia Appendix 3 summarizes the methodological quality assessment of RCTs. The risk of bias in the RCT was low.

The quality assessment for the only nonrandomized controlled trial shows a low risk of bias on preintervention, intervention and postintervention, and therefore, a low overall risk of bias [50].

### AVG Interventions

There was a great deal of variety across the AVGs used. Interventions mostly ran during physical education lessons, during playtime or lunch time as extracurricular activities after school or at home. The most commonly used devices in AVG interventions were gaming consoles, such as Xbox 360 with Kinect, Nintendo Wii, Sony PlayStation 2, dance mats, and interactive video game cycling. Games included Just Dance, Wii Fit, Wii Sports, Kinect Adventures, Kinect Sport, Dance Central, Dance Dance Revolution (DDR), and EyeToy.

The length of AVG interventions ranged from 8 weeks to 6 months (mean 16.3, SD 6.7 weeks). The frequency of AVG sessions ranged from 1 day to 5 days per week (mean 126.3, SD 55.8 minutes per week). Sessions typically lasted between 30 and 90 minutes (mean 52.0, SD 11.1 minutes) and were delivered by teachers and research assistants. It is therefore complicated to establish a standard length, intensity, and duration of sessions or type of the AVG intervention.

The different control groups either performed another intervention without AVGs, such as physical education or exercise sessions, access to sedentary video games, and learning sessions or were only asked to continue their normal activities of daily life, the latter being the most used option for the control group.

### AVG Effects

All the studies concerning the effects of AVG on motor competence and health-related physical fitness in children and adolescents with overweight and obesity are summarized in Multimedia Appendix 4.

A total of 15 randomized and nonrandomized controlled trials showed effects of AVGs on health-related physical fitness [42,44,51-62] and motor competence [50,61] in children and adolescents with overweight and obesity.

BMI, fat mass, or body fat percentage were measured in 14 studies using dual-energy x-ray absorptiometry [52,54] or bioelectrical impedance [51,58-60] to measure body fat. Waist circumference was measured in 4 studies [42,51,52,58]. CRF was evaluated in 8 studies using different tests, such as the 20-m shuttle run test [42,51,59,61], the 3-minute step test [57], and a submaximal test with a cycle ergometer [58,60]. Motor competence was only measured by Van Biljon et al [50] using the Bruininks-Oseretsky Test and by Bonney et al [61] using the Movement Assessment Battery for Children Test-Second Edition.

**Figure 2:**
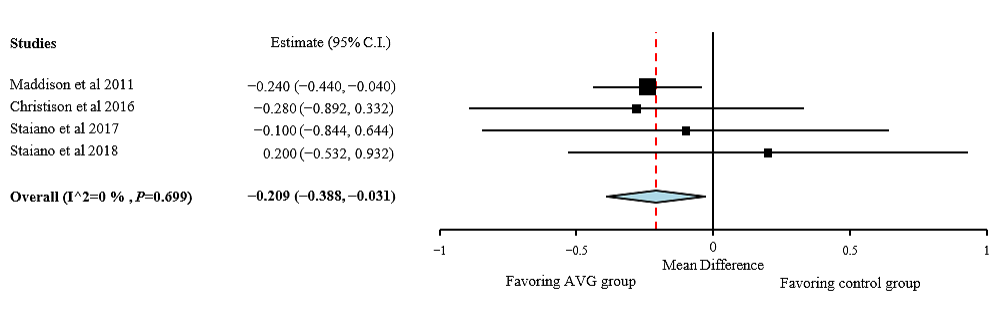
BMI effect sizes for active video games compared with those for control group. AVG: active video game.

**Figure 3:**
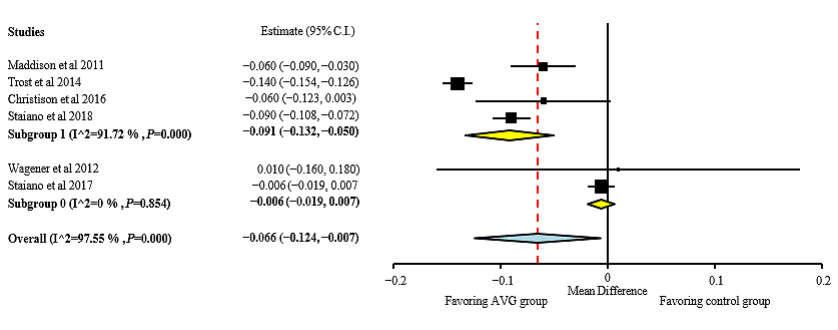
BMI z-score effect sizes for active video games compared with those for control group. Analysis by length of the intervention: subgroup 1: interventions lasting more than 12 weeks; subgroup 0: interventions lasting 12 weeks or less. AVG: active video game.

**Figure 4:**
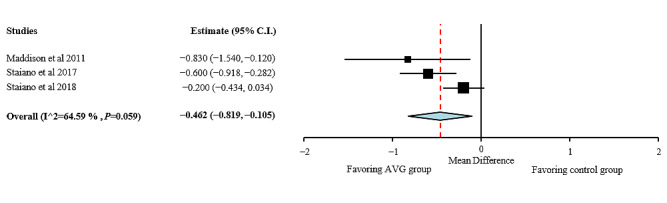
Body fat percentage effect sizes for active video games compared with those for control group. AVG: active video game.

**Figure 5:**
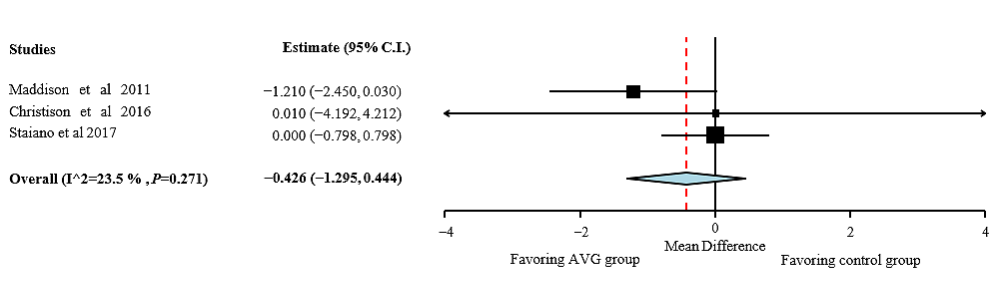
Waist circumference effect sizes for active video games compared with those for control group. AVG: active video game.

**Figure 6:**
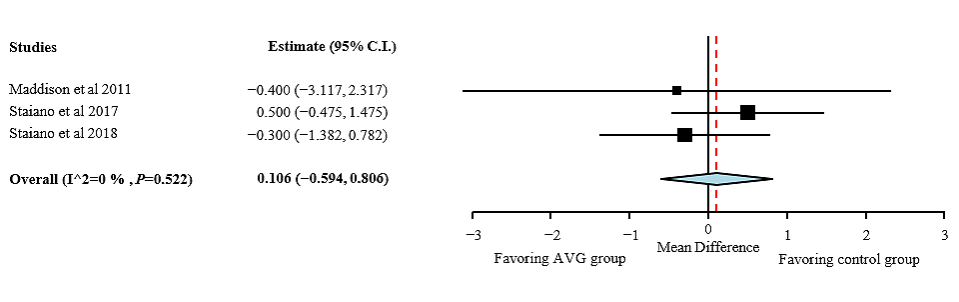
Fat-free mass effect sizes for active video games compared with those for control group. AVG: active video game.

A quantitative analysis was performed for BMI, BMI z-score, body fat percentage, fat mass, fat-free mass, and waist circumference. Individual study results and global effects are presented in Figures 2-6, whereas a summary of the global results is presented in Table 1. Data from 2 studies [51,54] were included, taking into consideration when interpreting the data that the results obtained by these studies were adjusted for baseline outcome measures, age, and sex. Before including them, it was ascertained that the studies did not change the trend of the results without them.

**Table 1:** Effect sizes and heterogeneity of findings for studies comparing active video game intervention versus control group (N=15).

Measures	Studies, n (%)	Hedge *g* effect size	Value, 95% CI	*P* value	I² (%)
BMI	4 (27)	−0.209	−0.388 to −0.031	.70	0
BMI z-score	6 (40)	−0.066	−0.124 to −0.007	<.001	97.55
Body fat percentage	3 (20)	−0.462	−0.819 to −0.105	.06	64.59
Fat-free mass	3 (20)	0.106	−0.594 to 0.806	.52	0
Waist circumference	3 (20)	−0.426	−1.295 to 0.444	.27	23.5

A quantitative analysis was not performed for CRF because of different measurement methods and articles with the same sample and for motor competence or muscular fitness because of the lack of articles. Some articles were excluded from the quantitative analyses given that the effect sizes could not be calculated from the information available in the papers [56,57], the sample was the same between studies [44,59], or the number of studies found was insufficient [50]. Another article was excluded from the quantitative analyses because of the control group exercised [58,60,61]. Noncontrolled trials [63-67] were considered in the discussion and are summarized in Multimedia Appendix 1.

#### Weight, BMI, and Body Fat

A total of 13 studies evaluated changes in weight, BMI, fat mass, or body fat percentage measured. Of the 13 studies, 9 reported positive effects of an AVG intervention on body weight, BMI, or body fat in children with overweight and obesity. The first study that investigated the effect of AVG on BMI status and body composition in adolescents with overweight and obesity was performed by Adamo et al [58], and they compared a 10-week AVG cycling intervention with a stationary bike music intervention. There were no significant group or group by time effects on body weight, BMI, fat mass, or fat-free mass, but a reduction in body fat percentage was found when groups were combined and compared with baseline. Nonsignificant results could be explained by the small sample size of the study and the shortness of the intervention period. The difference in effect sizes produced by the 2 types of training on BMI or body fat could be explained by the different energy expenditure, because AVG cycling intervention spent 576.2 kcal, whereas stationary bike music group spent 554.6 kcal.

Maddison et al [51] investigated the effect of the EyeToy of PlayStation on the body composition of 322 children. Participants in the intervention group were encouraged to meet the recommendations of 60 minutes per day and to substitute periods of traditional inactive video games, and they received a PlayStation 2 and the EyeToy to play at home. Differences between control and intervention groups were found for BMI (0.24 kg/m²; 95% CI −0.44 to −0.04; *P*=.02), BMI z-score (0.06; 95% CI −0.12 to −0.03; *P*=.03), body weight (0.72 kg; 95% CI −1.33 to −0.10; *P*=.02), fat mass (0.80 kg; 95% CI −1.36 to −0.24; *P*=.005), and body fat percentage (0.83%; 95% CI −1.54 to −0.12; *P*=.02), favoring the AVG group. A year later, derived from the previous study, the authors studied the mediating effect of CRF on body composition and concluded that an AVG intervention with EyeToy can have a positive effect on body composition in children with overweight or obesity and that this effect is most likely mediated through an improvement in CRF [59]. Foley et al [44] divided the analyses of the previous study by subgroups such as ethnicity, sex, and CRF level, and the results showed that AVG can be used to improve body composition regardless of ethnicity, sex, and CRF level.

Staiano et al [56] studied the effect of playing Nintendo Wii on school days on the weight of adolescents and compared the effects between co-operative and competitive AVGs versus a control group. The results showed that the co-operative AVG group lost more weight than the control group, whereas the competitive AVG group did not differ from the co-operative AVG group and control group. The authors studied the effect of psychological variables on weight loss and, as expected, those who had higher peer support at baseline lost marginally more weight over time, but, unexpectedly, initial self-efficacy did not affect weight change over time, nor did higher self-esteem cause more weight loss over time. The 2 studies by Staiano et al [52,54] investigated the effects of AVG interventions using Xbox Kinect. In the first study [52], a dancing AVG had no effects on or differences between the AVG and control groups. The small sample size and the short length of the intervention could explain the nonsignificant effects of AVG. In the other study by Staiano et al [54], a home-based AVG intervention led to a reduction in the BMI z-score (mean −0.06, SD 0.03) and the weight z-score (mean −0.09, SD 0.05) in the AVG group in comparison with the control group (mean 0.03, SD 0.03 and mean 0.07, SD 0.04 for BMI z-score and weight z-score, respectively) when one control outlier was excluded. There was a nonsignificant intervention effect for fat mass or body fat percentage. This could be influenced by the small sample size, in addition to the fact that the performance in the sessions with AVG at home showed lower or no benefits.

Trost et al [55] compared the effects of a 16-week weight management program with family-based theoretical sessions focused on lifestyle and the effects of the same program with AVG intervention. The overweight rate was reduced by 5.4% in the weight management program without PA and 10.9% in the AVG group, with significant pre-post and between-group differences. Both groups exhibited reductions in BMI z-score, but the AVG group showed greater reductions (mean −0.25, SD 0.03 vs mean −0.11, SD 0.03).

The most recent study was conducted by Irandoust et al [62], and the results showed reductions in weight and BMI in the AVG and exercise groups from pretest to posttest, resulting in lower weight and BMI at posttest measures in these groups in comparison with the control group after a 6-week intervention using Xbox Kinect and Nintendo Wii.

Of the 13 studies, 4 reported no positive effects of an AVG intervention on BMI or body fat in children with overweight and obesity [42,52,53,57]. Apart from Staiano et al [52], no other authors found effects after an AVG intervention. Christison et al [42] compared the effects of weight management didactic sessions with an AVG intervention with Nintendo Wii and DDR. The results showed a trend of reduction in the BMI z-score in the AVG group, whereas the BMI z-score among the control group was essentially unchanged (*P*=.07). Wagener et al [53] showed no pre-post differences in BMI within or between conditions after 10 weeks of an AVG intervention by playing dance-based AVGs. Probably, no benefits of AVGs and even nonsignificant worse results on BMI for the AVG group were found because of the small sample size and the short length of intervention. There were same limitations in the study performed by Maloney et al [57], which showed that there were no changes between pretest and posttest participant weight in the AVG group or control group after 12 weeks of intervention with AVGs.

Furthermore, 5 noncontrolled studies observed positive effects of an AVG intervention on BMI or body fat in children with overweight and obesity. The most recent study was performed by Argarini et al [67], and the results showed a significant decrease in weight, BMI, and body fat percentage after a 4-week AVG intervention with Xbox Kinect. Christison et al [66] evaluated the efficacy of an AVG intervention and the results showed a significant decrease in BMI (mean −0.48, SD 0.93 kg/m²) and BMI z-score (mean −0.07, SD 0.14) after 10 weeks of training (*P*=.002 and *P*<.001, respectively). Duman et al [64] investigated the effects of a combination of music-accompanied aerobics, callisthenic exercises, and AVGs, and the results showed decrease in BMI and triceps skinfold thickness. The percentage of obese children decreased from 72% to 40%; those children who were obese became children with overweight, so the percentage of overweight children increased from 28% to 46%; the percentage of children with normal weight increased from 0% to 14%. Calcaterra et al [65] demonstrated the effectiveness of a combination of circuit-based aerobics and strength and resistance exercises with AVGs, showing a significant decrease in BMI (from 32.9 to 31.9 kg/m²; *P*=.002) and body fat percentage (from 39.3% to 36.0%; *P*=.001). A very interesting result of this study was that 27.2% of the participants reported a previous negative experience with exercise, so a reduced drop-out rate during activity may be achieved with a playful aspect and adapted activities such as AVGs. Finally, Huang et al [63] investigated the effect of AVGs using Nintendo Wii and Xbox Kinect, with no effects on the percentage of body fat, probably because of the short length of the intervention and the reduced number of participants.

Systematic reviews have been performed on the effects of AVG on BMI or body fat, but they are mostly not focused on children or adolescents with overweight or obesity and including studies with children with normal weight; some limitations can be found in these studies, such as the inclusion of noncontrolled trials. The results of these studies are in line with the results of this study. The latest systematic review was performed by Gao et al [68], who included noncontrolled trials and studies with children with normal weight. Reduction in BMI after AVG interventions was found in children and adolescents. Hernández-Jimenez et al [69] performed a meta-analysis that showed a significant effect in favor of AVGs on BMI in children and adolescents, with better results achieved when the AVG intervention was applied to children with overweight or obesity. Another systematic review [70] included 4 RCTs, which are also included in this systematic review, which reported decreases in BMI or body fat after an AVG intervention. A previous systematic review performed by Gao et al [31] concluded that AVGs were a promising tool to promote PA and health as long as the AVG intervention is not home based, but this review did not focus on children with overweight or obesity. Two systematic reviews [30,71] supported the findings, although being among the first reviews on the effects of AVGs, quantitative analyses were not conducted because of a lack of articles. Lamboglia et al [30] found that AVG led to increased PA and CRF and decreased body fat, with considerable potential to fight obesity. Leblanc et al [71] found that AVG attenuated weight gain in participants with overweight and obesity, including 3 articles that are included in this systematic review. The improvement of cardiometabolic health through AVG was inconclusive because of the small number of articles at the time.

#### Quality Assessment of BMI, Body Fat Percentage, and Fat Mass

As shown in Figure 2, positive effects of the interventions were found for BMI, favoring the AVG group compared with the control group with no intervention (MD −0.209; 95% CI −0.388 to −0.031). Heterogeneity among studies for BMI was low (I²=0%; *P*=.70). AVG showed more positive effects on BMI z-score than on BMI (MD −0.066; 95% CI −0.124 to −0.01), but it also showed a very high heterogeneity (I²=97.55%; *P*<.001; Figure 3). The results of the subgroup analysis by the length of the intervention showed that the decrease in BMI z-score was higher in the AVG interventions longer than 12 weeks.

As shown in Figure 4, positive effects of AVG interventions were found for body fat percentage, favoring the AVG group compared with the control group with no intervention (MD −0.462; 95% CI −0.819 to −0.105). Heterogeneity among studies for BMI was high (I²=64.59%; *P*=.06).

These results clearly showed the influence of AVG intervention length on weight, BMI, and body fat percentage. Positive effects were observed for AVG interventions longer than 12 weeks. It seems that a combination of AVG with multicomponent exercises could have more benefits on BMI and body fat percentage in children and adolescents with overweight and obesity, but RCTs are needed to confirm this.

#### Waist Circumference

Changes in waist circumference were evaluated by 4 RCTs [42,51,52,58], and no effects were found. The first study was performed by Adamo et al [58] and showed no effects or differences between groups for waist circumference. Maddison et al [51] reported no changes in waist circumference after a 24-week AVG intervention with EyeToy performed at the participants’ homes. Christison et al [42] performed a 6-month AVG intervention with Nintendo Wii and DDR; they also did not report positive effects or differences between groups. The most recent study on waist circumference in children and adolescents with overweight and obesity was performed by Staiano et al [52], who investigated the effects of Xbox Kinect and found no effect on waist circumference or differences between AVG and control groups.

The noncontrolled trial performed by Calcaterra et al [65] demonstrated a decrease in waist circumference (-5.9 cm) and waist circumference to height ratio (-0.08) after a 12-week training program combining traditional exercise with AVGs.

#### Quality Assessment of Waist Circumference

As shown in Figure 5, no overall effects were found on waist circumference after the AVG interventions (MD −0.426; 95% CI −1.295 to 0.444). Heterogeneity among studies for waist circumference was moderate (I²=23.5%; *P*=.27). AVGs seem not to be effective in decreasing waist circumference in children and adolescents with overweight and obesity. It is necessary to look for a way to increase the demands of the activity.

Interventions with AVG do not seem to be effective in decreasing waist circumference in children and adolescents with overweight and obesity. This result may be because of the length of the interventions in the included articles. A reduction in the waist circumference in children with obesity seems to be possible with a combination of AVG with multicomponent exercise instead of AVG exclusively, but RCTs are needed to confirm this. Waist circumference is as important as BMI or body fat percentage because they are good predictors of cardiovascular disease risk factors in children and adolescents, even better than BMI [72]. Therefore, the main aim is to decrease waist circumference of children and adolescents with overweight and obesity. However, the results suggest that AVG interventions do not seem to be effective in decreasing waist circumference in children and adolescents with overweight and obesity.

#### Fat-Free Mass

The effects of AVG on fat-free mass were reported in 4 articles [51,52,54,58]. To measure fat-free mass, bioelectrical impedance [51,58] or dual-energy x-ray absorptiometry [52,54] were used. Maddison et al [51] did not find any effects or differences between groups for fat-free mass. Similar results were reported by Adamo et al [58], with no changes or differences between groups. In contrast, Staiano et al [52,54] reported no effects on lean mass after an AVG intervention. Evidence on the effect of AVGs on fat-free mass is limited, and no effects have been shown.

#### Quality Assessment of Fat-Free Mass

As shown in Figure 6, no overall effects were found for fat-free mass after the AVG interventions (MD 0.106; 95% CI −0.594 to 0.806). Heterogeneity among studies for waist circumference was low (I²=0%; *P*=.52).

#### Cardiorespiratory Fitness

CRF assessments following their AVG interventions were included by 5 studies, and 4 of them managed to find positive results. In general, CRF was improved after an intervention with AVG, such as Nintendo Wii, DDR, EyeToy, or a cycling AVG.

The first study that reported the effects of AVG on CRF in adolescents with overweight and obesity was the study by Adamo et al [58], who observed a significant training effect over time in both AVG cycling and stationary bike interventions. Both interventions produced significant improvements in peak heart rate, peak workload, and time to exhaustion, but no significant differences were found between the exercise groups. With this same intervention, Goldfield et al [60] observed that the psychological benefits of aerobic exercises were related to improved aerobic fitness. The abovementioned study by Maddison et al [51] did not find significant increases in CRF in the AVG group, but the positive effect of AVG on body composition in children with overweight or obesity is most likely mediated through improved aerobic fitness [59]. Maloney et al [57] showed no improvements in CRF in either the AVG or control group after playing DDR for 12 weeks. Christison et al [42] showed that the number of shuttle runs did not change after a 6-month AVG intervention. The most recent study was conducted by Bonney et al [61], who investigated the effect of Wii Fit in comparison with a task-oriented functional training on the performance of the shuttle run test and positive effects on CRF in both groups, but no differences were found between the AVG and control groups performing the task-oriented functional training.

The effects of AVG on CRF in children with overweight and obesity have been studied by 2 noncontrolled trials [63,65]. Calcaterra et al [65] demonstrated in their study an improvement in CRF (3.8 mL/kg/min; *P*<.001) measured by a walking test on a treadmill reaching 85% of the maximal heart rate. Huang et al [63] showed no effects of AVG using Nintendo Wii and Xbox Kinect on CRF, but the heart rate demonstrated that most participants were able to achieve moderate or vigorous intensity of exercise during most AVG sessions.

The effect of AVG interventions on CRF remains unclear. Probably, the limited effects of AVG interventions on CRF of children with overweight and obesity might be because of insufficient training volume in terms of either weekly frequency or overall duration of the interventions. As mentioned earlier, interventions performed at home could be ineffective for improving health-related physical fitness, such as CRF. As it occurs in children with normal weight, only Calcaterra et al [65] used a submaximal or maximal incremental cardiopulmonary exercise test with a gas analyzer, which is widely recognized as the best single index of aerobic fitness [73,74]. Once again, science-based evidence shows that a combination of AVG with multicomponent exercise could produce more benefits on CRF than AVG exclusively, probably because of a higher volume of training. Therefore, these results must be interpreted with caution because the studies that report results from interventions using AVGs with multicomponent exercise are noncontrolled trials. RCTs are needed to confirm this finding.

A systematic review performed by Zeng and Gao [70] included only 1 RCT, also included in this systematic review, which reported positive effects of an AVG intervention in comparison with the effects of an exercise group, but these results were unclear because of the inclusion of only 1 article.

#### Muscular Fitness

Only 1 RCT [61] and 3 noncontrolled trials [63-65] investigated the effects of an AVG intervention on the muscular fitness of children and adolescents with overweight and obesity. The only RCT about the effects of AVGs on muscle fitness showed that both the AVG group trained with Wii Fit and the control group that performed a task-oriented functional training for 14 weeks improved knee extensors and ankle plantar flexors to maximal isometric strength assessed with a handheld dynamometer.

Calcaterra et al [65] demonstrated an increase in muscular strength, improving from a mean of 29.6 kg (SD 9.3 kg) to 32.3 kg (SD 9.8 kg; *P*=.003) in a handgrip test. Duman et al [64] investigated the effects of an AVG intervention combined with traditional exercise on several physical performance tests that require muscle strength and endurance, such as time to ascend and descend 20 stairs, number of squats they can perform in 120 seconds, time to run 50 m, and rope jumps in 30 seconds. The results showed enhancements in all test performances. Finally, Huang et al [63] investigated the effect of AVG using Nintendo Wii and Xbox Kinect on the muscular strength of the quadriceps and hamstrings that were assessed using a handheld dynamometer and muscular endurance that were assessed by a 1-minute half-sit-up test consisting of completing as many half-sit-ups as possible within a minute. Nonsignificant changes in the muscular strength of the quadriceps or muscular endurance were observed. Low frequency and program duration may explain the lack of significant changes.

A combination of AVG with multicomponent exercise could enhance muscular fitness in children with overweight and obesity, but RCTs are required to confirm these results. A systematic review and meta-analysis [5] showed the importance of muscular fitness for children and adolescents and found associations between muscle fitness and bone health, total and central adiposity, and cardiovascular diseases, metabolic risk factors and self-esteem. In addition, according to Tomlinson et al [75], children and adolescents with overweight and obesity have a greater absolute maximum muscle strength than nonobese persons because increased adiposity induces a chronic overload stimulus on the antigravity muscles; however, when maximum muscular strength is normalized to body mass, individuals with obesity appear weaker, probably because of reduced mobility, neural adaptations, and changes in muscle morphology. Therefore, it is important to include exercises aimed at improving muscular fitness in programs for children and adolescents with overweight and obesity.

#### Motor Competence

Motor competence after an AVG intervention was reported in 2 articles. The first was performed by Van Biljon et al [50], and motor competence was evaluated in 30 individuals with overweight and obesity using the shorter version of the Bruininks-Oseretsky test for motor proficiency. The intervention group performed a 6-week AVG intervention for 3 days per week and 30 minutes per session using Wii; there were 2 control groups, one with access to traditional video games and the other continued with their everyday life activities with no intervention. The AVG group showed improvements in motor competence compared with both control groups, specifically in terms of agility and speed, co-ordination, and reaction time. Another more recent study by Bonney et al [61] showed that both the AVG group that trained with Wii Fit and the control group that performed a task-oriented functional training for 14 weeks improved motor co-ordination, as measured by the Movement Assessment Battery for Children Test-Second Edition. A notable difference between the controlled trials investigating the effect of AVG on motor competence is the length of the intervention; therefore, the study with the longest duration of the intervention [61] (14 weeks) showed positive effects, whereas the one with the shortest duration [50] (6 weeks) showed no effect after the AVG intervention. Another study with no control group showed an improvement in motor competence after 4 weeks of training with Xbox Kinect [67]. This scarcity of studies investigating the effect of AVG on the motor competence of children and adolescents with overweight and obesity is of great importance, given that evidence shows a relationship between motor competence and health-related physical fitness during childhood and adolescence [20], as mentioned earlier. Furthermore, low motor competence, denominated as *physical illiteracy*, is a component of the pediatric inactivity triad observed by Faigenbaum et al [76], together with exercise deficit disorder and pediatric dynapenia. These 3 components are closely related to each other. Children and adolescents who perceive themselves as having poor motor competence might feel less inclined to participate in PA or sports, which in turn will reduce their ability to improve their muscular fitness or motor competence. Motor competence is particularly important in children and adolescents with overweight and obesity, as they have a lower motor competence level than children and adolescents with a healthy weight [16]; this has health consequences because it is directly related to PA. Improving motor competence could increase PA, reduce sedentary behaviors, and positively impact health-related physical fitness [16,77]. Improvements in motor competence entail higher motivation and participation in extracurricular PA and sports [18,19]. AVGs could be a tool for improving motor competence, but randomized clinical trials are needed to corroborate this.

Interventions with AVG appear to be more effective in decreasing BMI and body fat in children and adolescents with overweight or obesity than in children with normal weight, but effectiveness in other health-related physical fitness parameters such as waist circumference or CRF is still unclear. Children and adolescents with overweight or obesity could benefit from AVGs to improve their motor competence, which seems to be a variable more susceptible to enhancement as improvements have been observed with shorter interventions, but further research is needed to confirm this hypothesis. Therefore, AVG programs could be a good strategy to combat childhood obesity.

## Discussion

### Principal Findings

This paper provides knowledge about the effects of AVG on health-related physical fitness and motor competence in children and adolescents by gathering previous scientific evidence, and it also provides the prospects for future studies.

Mental health can be considered as a major influencing variable and can undoubtedly influence the effects of AVG interventions on childhood obesity; however, this systematic review has focused on the effects of AVG interventions on health-related physical fitness and motor competence in children and adolescents with overweight and obesity, discarding the psychological aspects. However, a very recent overview [37] and a systematic review [36] found a positive effect of AVG on mental health, but it also showed the need for increasing scientific research in this area.

In contrast, the participants in the studies included in this review were from countries with a medium-to-high socioeconomic status; this leads to the limitation that such AVG interventions are difficult to implement or are not applicable in countries with a low socioeconomic status because of the lack of resources and lower purchasing power. It would be interesting to study the feasibility and possibilities of such interventions in societies with low socioeconomic status.

Furthermore, studies investigating the effects of AVG in children with obesity with some information from geohash dashboards related to the environment where the participants live and where the school is located can be useful to increase the strength of the hypothesis on how AVG affects the features addressed, as there are several features where weak correlation has been found with AVG use, as. As stated earlier, it is necessary to establish the guidelines for an effective intervention using AVGs. It seems that the most effective application of an AVG intervention is a sufficient duration and the structuring and planning of that intervention. In addition, most of the scientific evidence that has studied AVG interventions combined with traditional exercise lacked a control group to compare the effects; these AVG interventions seem to be promising, but RCTs are needed to investigate the effects of these AVG interventions.

Finally, few studies have examined the effects of an AVG intervention on muscular fitness and motor competence, which, as mentioned earlier, are components of the pediatric inactivity triad observed by Faigenbaum et al [76] together with exercise deficit disorder. The importance of these 2 variables lies in their close relationship with PA and sports participation, which can improve the physical fitness and body composition of children and adolescents, which is especially important in those who are overweight or obese. By improving the muscle strength and motor skill of these children and adolescents, we may make them more active and therefore healthier.

RCTs are needed to investigate the effects of AVG interventions on children's muscle strength and motor skills to learn about the possibilities of AVG interventions in stopping the vicious circle of pediatric inactivity triad.

### Limitations

The limitations of this review should be acknowledged. A wide variety of AVG interventions have been included, with different devices and training interventions (duration, frequency, training setting or training dynamic, and type of AVG), which makes it difficult to analyze all the articles together and to obtain generalized results. In addition, the potential risk of bias of some studies was not considered when interpreting the results. Finally, some subgroup analyses were not performed because of the small number of controlled trials. Gender, demographics, or race influence were not deeply addressed because the studies did not show the results divided by these covariates. However, it would be interesting to investigate whether such interventions are more effective depending on them.

### Strengths

This study also has several strengths. To the best of our knowledge, this is the first meta-analysis to summarize the existing research on the effects of AVG on health-related physical fitness and motor competence in children and adolescents with overweight and obesity. This analysis included not only the effects of AVG on BMI, but also those on body composition, CRF, muscular fitness, and motor competence. This study allowed us to realize that more RCTs reporting motor competence and muscular fitness results are needed.

### Conclusions

AVGs could be a good strategy to fight childhood obesity. AVG programs showed positive effects on BMI and body fat percentage. Improvements in CRF have been observed after an AVG intervention. Children and adolescents could benefit from AVGs to improve motor competence, but further research is needed to confirm these results. The effects of AVG programs on muscular fitness or fat-free mass are also unclear.

In conclusion, AVGs seem to be an effective tool to improve health-related physical fitness and is a promising tool for improving motor competence in children and adolescents. AVGs can even be considered as a prospective alternative to traditional exercise for enhancing health status during childhood.

## Appendix

Multimedia Appendix 1 PRISMA (Preferred Reporting Items for Systematic Reviews and Meta-Analyses) 2020 item Checklist.

Multimedia Appendix 2 Descriptive characteristics of included noncontrolled trials.

Multimedia Appendix 3 Quality assessment of randomized controlled trials.

Multimedia Appendix 4 Descriptive characteristics of included studies with children with overweight and obesity.

## Abbreviations

AVG: active video game
CRF: cardiorespiratory fitness
DDR: Dance Dance Revolution
MD: mean difference
PA: physical activity
PICOS: Population, Intervention, Comparison, Outcomes and Study
PRISMA: Preferred Reporting Items for Systematic Reviews and Meta-Analyses
RCT: randomized controlled trial
ROBINS-I: Risk of Bias in Nonrandomized Studies-of Interventions
