# Supplementary material for: Effects of Active Video Games on Health-Related Physical Fitness and Motor Competence in Children and Adolescents With Overweight or Obesity: Systematic Review and Meta-Analysis
Source: JMIR Serious Games. 2021 Oct 18;9(4):e29981. doi: 10.2196/29981 (PMC8561411; doi:10.2196/29981)
Supplement: Multimedia Appendix 2 [file games_v9i4e29981_app2.pdf]

Table S2: Descriptive characteristics of included non-controlled trials

Study	Participants		Intervention	Training	Variables and test used	Outcomes
	N	Years				
Argarini et al., 2020 [67]	n= 17<br>male (7)<br>female (10)	8.30 ± 1.55	Xbox Kinect	Period: 4 weeks<br>Frequency: 3 sessions per week<br>Duration: 30-40 min[i] per session	Body weight<br>BMI[c]<br>%BF[a] (Skin thickness)<br>MC[g] (catch, vertical jump and kick)	A significant improvement in vertical jump was observed. There was also significant decline on body weight, BMI, and %BF.
Huang et al., 2017 [63]	n= 10<br>male (8)<br>female (2)	8.0 ± 1.8	Nintendo Wii and Xbox Kinect	Period: 8 weeks<br>Frequency: two sessions per week<br>Duration: 60 min per session	BMI<br>%BF<br>MF[h] (one-minute half sit-up test and handheld dynamometer: quadriceps in seated position and hamstrings prone position)<br>CRF[d] (20-m shuttle run test)	There were improvements in quadriceps muscle strength, muscle endurance test (sit-ups) and flexibility. The body composition showed a slight improvement during the midterm of the program, but the effect was not maintained throughout the program. There was no significant change on hamstring muscle strength and CRF.

					Flexibility (sit and reach)	
Duman et al., 2016 [63]	n= 50<br><br>male (21)<br>female (29)	12.16 ± 0.99	TE[J]+AVG[b]: Callisthenic and aerobic exercises for 20 min + AVG for 25 minutes	Period: 8 weeks<br><br>Frequency: 3 days per week	BMI<br><br>FM[f] (triceps skinfold thickness)<br><br>Waist circumference<br><br>CRF<br><br>MF (50-m run, squats in 120 seconds, time up and down 20 stairs and jumps over a rope in 30 seconds)	TE+AVG showed decreases in BMI, FM and waist circumference; TE+AVG showed higher CRF and MF (performance in all tests)
Calcaterra et al., 2013 [65]	n= 22<br><br>male (13)<br>female (9)	13.23 ± 1.76	EXE[e]+AVG: a combination of circuit-based aerobics, strength and resistance exercises with interactive video game exercises	Period: 12 weeks<br><br>Frequency: twice a week<br><br>Duration: 90 min per session	BMI<br><br>Waist circumference<br><br>%BF (bioelectrical impedance)<br><br>CRF (during a submaximal	BMI, waist circumference and %BF decreased significantly; CRF and MF improved significantly.

					treadmill test)<br><br>MF (Hand-grip dynamometry)	
Christison et al., 2012 [66]	n= 40<br><br>male (26)<br>female (22)	11.2 ± 2.2	DDR; Exerbike XG ; Nintendo Wii; Makoto Interactive Arena; Lightspace Play Floor; Cybex Trazer; Treadwall; Xavix system	Period: 10 weeks<br><br>Frequency: 2h per week	BMI	BMI and BMI z-score decreased significantly

[a]%BF: body fat percentage, [b]AVG: active video game, [c]BMI: body mass index, [d]CRF: cardiorespiratory fitness, [e]EXE: exercise, [f]FM: fat mass, [g]MC: motor competence, [h]MF: musculoskeletal fitness, [i]min: minutes, [j]TE: traditional exercise.
