# Supplementary material for: Effects of Active Video Games on Health-Related Physical Fitness and Motor Competence in Children and Adolescents With Overweight or Obesity: Systematic Review and Meta-Analysis
Source: JMIR Serious Games. 2021 Oct 18;9(4):e29981. doi: 10.2196/29981 (PMC8561411; doi:10.2196/29981)
Supplement: Multimedia Appendix 3 [file games_v9i4e29981_app3.pdf]

**Table 1:** Quality assessment of randomized controlled trials.

| Study | Bias arising from the randomization process | Bias due to deviations from intended interventions | Bias due to missing outcome data | Bias in measurement of the outcome | Bias in selection of the reported result | Overall risk of bias |
|---|---|---|---|---|---|---|
| Irandoust et al (2020) [62] | + | + | + | + | + | + |
| Bonney et al (2019) [61] | + | + | + | + | + | + |
| Staiano et al (2018) [54] | + | + | + | + | + | + |
| Staiano et al (2017) [52] | + | + | + | + | + | + |
| Christison et al (2016) [42] | ? | + | ? | + | + | + |
| Foley et al (2014) [44] | + | + | + | + | + | + |
| Trost et al (2014) [55] | ? | + | ? | + | + | + |

| | | | | | | |
|---|---|---|---|---|---|---|
| Staiano et al (2013) [56] | + | + | ? | + | + | + |
| Maloney et al (2012) [57] | + | + | + | + | + | + |
| Goldfield et al (2012) [60] | + | + | ? | + | + | + |
| Maddison et al (2011) [51] | + | + | + | + | + | + |
| Maddison et al (2011) [59] | + | + | + | + | + | + |
| Adamo et al (2010) [58] | + | + | + | + | + | + |
