# Supplementary material for: Effects of Active Video Games on Health-Related Physical Fitness and Motor Competence in Children and Adolescents With Overweight or Obesity: Systematic Review and Meta-Analysis
Source: JMIR Serious Games. 2021 Oct 18;9(4):e29981. doi: 10.2196/29981 (PMC8561411; doi:10.2196/29981)
Supplement: Multimedia Appendix 4 [file games_v9i4e29981_app4.pdf]

Table 2: Descriptive characteristics of included studies with overweight and obese children.

Study	Participants			Study design	Intervention	Control	Training	Variables and test used	Outcomes
	Population, N	Sex	Age (years)						
Irandoust et al (2020) [62]	59	Male (n=59)	Primary school (6-11 years)	RCT[a]	Nintendo Wii and Xbox Kinect (n=21; mean 8.9, SD 1.2 years)	CG[b] 1: aquatic exercise intervention (n=18; mean 9.3, SD 1.3 years); CG 2: no intervention (n=20; mean 8.95, SD 1.2 years)	Period: 12 weeks; frequency: 3 days per week; duration: 60 min per session	Weight and BMI	AVG[c] and CG 1 observed decreased weight and BMI compared with CG 2.
Bonney et al.(2019) [61]	52	Female (n=52)	13-16 years	RCT	Wii Fit (n=26)	Task-oriented functional training (n=26)	Period: 14 weeks; frequency: once a week; duration:	CRF[d] (20-min shuttle run test); MF[e] (knee extensors, ankle plantar flexors	Both AVG and CG demonstrated significant improvement in

							45 min per session	isometric strength with a handheld dynamometer); and MC[f] (Movement ABC-2[g])	CRF, MF, and MC. No between-group differences were observed.
Staiano et al (2018) [54]	45	Male (54%); female (46%)	Mean 11.2, SD 0.8 years	RCT	Xbox Kinect (n=22)	No intervention (n=23)	Period: 24 weeks; frequency: 3 days per week; duration: 60 min per session	BMI; %BF[h]; and FM[i] (DXA[j])	Positive effects were observed on BMI z-score and weight z-score for AVG but not on FM and %BF.
Staiano et al (2017) [52]	41	Female (n=41)	14-18 years	RCT	Xbox Kinect (n=22; mean 15.3, SD 1.2 years)	No intervention (n=19; mean 16.1, SD 1.4 years)	Period: 12 weeks; frequency: 60 min per week	BMI; WC[k]; and FM and %BF (DXA)	No effects on BMI, WC, FM, and %BF were observed.
Christison et al	80	Male (n=34);	Mean 10.1, SD 1.3 years	RCT	Nintendo Wii, DDR[l], Exerbike	No intervention (only didactic session; n=21)	Period: 10 weeks;	BMI; WC; and CRF (20-min shuttle run)	No effects of intervention on BMI, WC, and

(2016) [42]		female (n=46)			XG, Makoto interactive Arena, Lightspace Pay floor, Cybex Trazer, and Zavix system (n=59)		frequency: 2 hours per week		CRF were observed.
Foley et al (2014) [44]	322	Male (n=235); female (n=87)	Mean 11.6, SD 1.1 years	RCT	EyeToy (PlayStation; n=160)	No intervention (n=162)	Period: 12 weeks and 24 weeks; children were encouraged to meet the recommendations of 60 min per day and to substitute periods of traditional	BMI; FM; and %BF (bioelectrical impedance)	Positive effects on BMI, z-score BMI, and %BF for AVG were observed.

							inactive video games		
Trost et al (2014) [55]	69	Male (n=24); female (n=41)	Mean 10.0, SD 1.7 years	RCT	CPWMP[m] with AVG with Xbox Kinect (n=31)	CPWMP only (n=38)	Period: 8 weeks and 16 weeks; frequency and duration: nonreported	BMI and BMI z-score	Groups with AVG showed higher decreases in BMI, BMI z-score, and overweight rate than CG.
Staiano et al (2013) [56]	54	Male (44.4%); female (55.6%)	15-19 years	RCT	Competitive AVG with Nintendo Wii (n=19); co-operative AVG with Nintendo Wii (n=19)	No intervention (n=16)	Period: 10 weeks and 20 weeks; frequency: 30-60 min per school day	Weight	Participants in co-operative AVG lost more weight than CG; competitive AVG also lost weight.

Maloney et al (2012) [57]	65	Male (n=31); female (n=34)	9-17 years	RCT	DDR (n=32; mean 12.9, SD 2.36 years)	No intervention (n=33; mean 11.73, SD 2.38 years)	Period: 12 weeks; frequency and duration: nonreported	Weight and CRF (3-min step test)	No effects on weight and CRF were observed.
Van Biljon et al (2012) [50]	31	Male and female	9-12 years	Non-RCT	Nintendo Wii (n=11)	CG1: with access to sedentary video games (n=10); CG2: no intervention (n=10)	Period: 6 weeks; frequency: 3 days per week; duration: 30 min per session	MC (Bruininks-Oseretsky Test)	Improvements in MC for AVG compared with both CGs were observed.
Wagener et al (2012) [53]	40	Male (n=31; female (n=34)	Mean 14, SD 1.66 years	Non-RCT	Dance-based exergaming (n=21)	No intervention (n=19)	Period: 10 weeks; frequency: 3 days per week; duration: 40 min per session	BMI z-score	No changes in BMI z-score were observed.
Goldfield et al (2012) [60]	26	Male (n=12; female (n=14)	12-17 years	RCT	Interactive video game cycling intervention	Exercise: stationary bike music intervention (n=13;	Period: 10 weeks; frequency: twice per week;	BMI; %BF and FFM[n] (bioelectrical impedance); and	AVG and CG showed improvements in CRF and %BF;

					(Gamebike; n=13; mean 15.1, SD 1.8 years)	mean 13.9, SD 1.4 years)	duration: 60 min per session	CRF (submaximal aerobic fitness with a cycle ergometer)	no group by time effects on body weight, BMI, FM, FFM, %BF, or CRF; psychological benefits of these aerobic exercise were related to improved aerobic fitness but not to changes in body composition.
Maddison et al (2011) [59]	322	Male (n=235); female (n=87)	Mean 11.6, SD 1.1 years	RCT	EyeToy (PlayStation; n=160)	No intervention (n=162)	Period: 12 weeks and 24 weeks; children were encouraged to meet 60 min per day and to substitute periods	BMI; %BF (bioelectrical impedance); and CRF (20-min shuttle test)	AVG positively affected %BF. This effect was most likely mediated through improved CRF.

							of traditional inactive video games		
Maddison et al (2011) [51]	322	Male (n=235); female (n=87)	Mean 11.6, SD 1.1 years	RCT	EyeToy (PlayStation; n=160)	No intervention (n=162)	Period: 12 weeks and 24 weeks; children were encouraged to meet the recommendations of 60 min per day and to substitute periods of traditional inactive videogame	BMI; WC; %BF (bioelectrical impedance); and CRF (20-min shuttle test)	Positive effect on BMI, BMI z-score, FM, and %BF was observed, favoring AVG.
Adamo et al (2010) [58]	26	Male and female	12-17 years	RCT	Interactive video game cycling intervention (n=13; mean	Exercise: stationary bike music intervention (n=13; mean 13.9, SD 1.4 years)	Period: 10 weeks; frequency: twice per week; duration: 60 min per session	BMI; %BF and FFM (bioelectrical impedance); WC; and CRF (submaximal	AVG and CG showed improvements in CRF; no group by time effects on body weight,

					15.1, SD 1.8 years)			aerobic fitness with a cycle ergometer)	BMI, FM, FFM, %BF, or CRF; positive time effects were found on %BF when both AVG and CG were combined and compared at baseline.

[a]RCT: randomized controlled trial.
[b]CG: control group.
[c]AVG: active video game.
[d]CRF: cardiorespiratory fitness.
[e]MF: musculoskeletal fitness.
[f]MC: motor competence.
[g]Movement ABC-2: Movement Assessment Battery for Children-Second Edition.
[h]%BF: body fat percentage.
[i]FM: fat mass.
[j]DXA: dual-energy x-ray absorptiometry.
[k]WC: waist circumference.
[l]DDR: Dance Dance Revolution.
[m]CPWMP: Comprehensive Pediatric Weight Management Program.
[n]FFM: fat-free mass.
